# Supplementary figures and images for: Gene length as a regulator for ribosome recruitment and protein synthesis: theoretical insights
Source: Sci Rep. 2017 Dec 12;7:17409. doi: 10.1038/s41598-017-17618-1 (PMC5727216; doi:10.1038/s41598-017-17618-1)

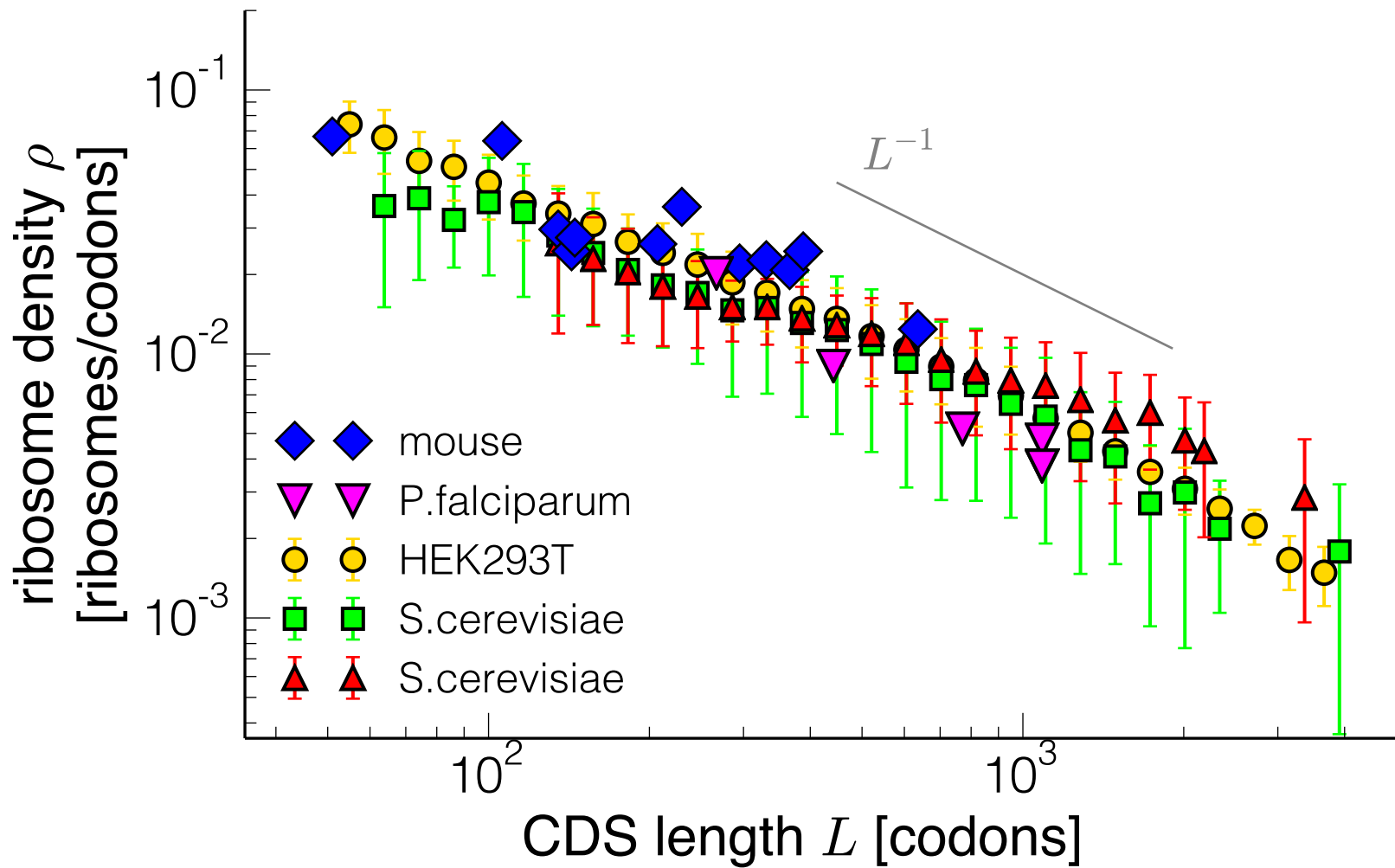

Supplement: Supplementary file 1 — LaTeX Supplementary File [file 41598_2017_17618_MOESM1_ESM.pdf]

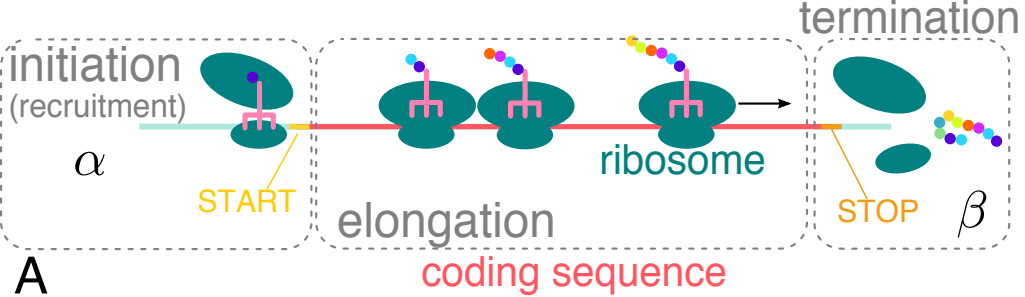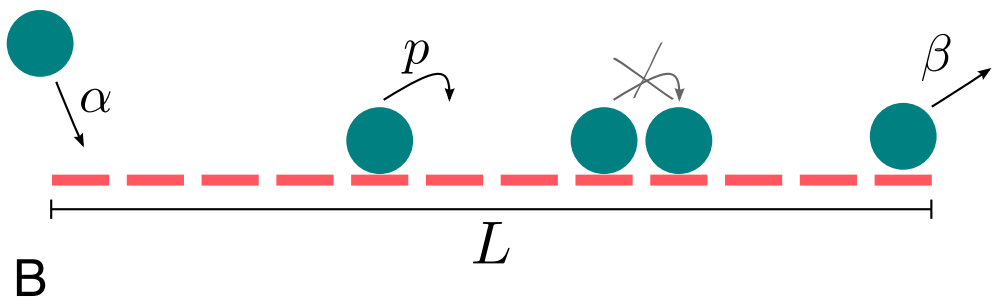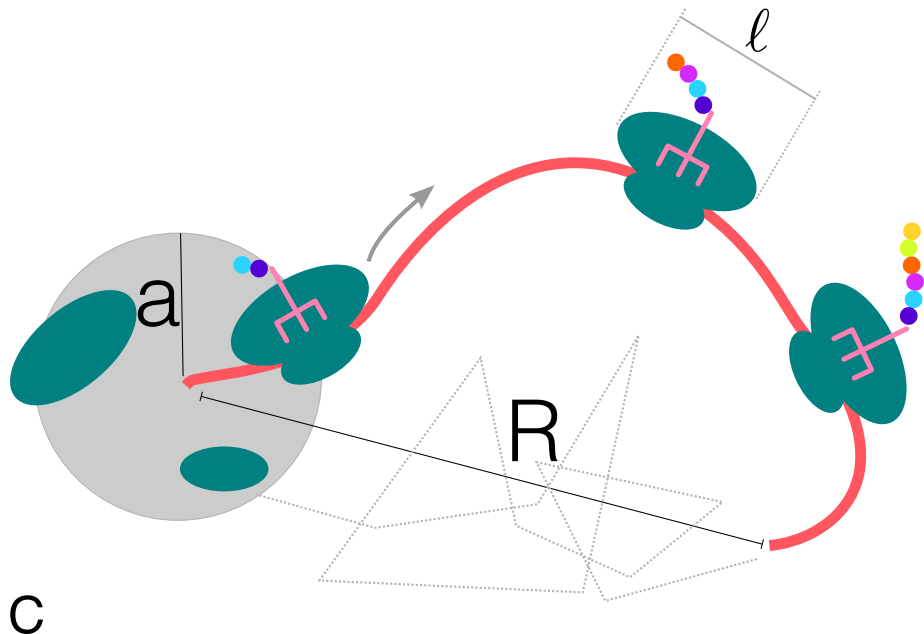

Supplement: Supplementary file 2 — LaTeX Supplementary File [file 41598_2017_17618_MOESM2_ESM.pdf]

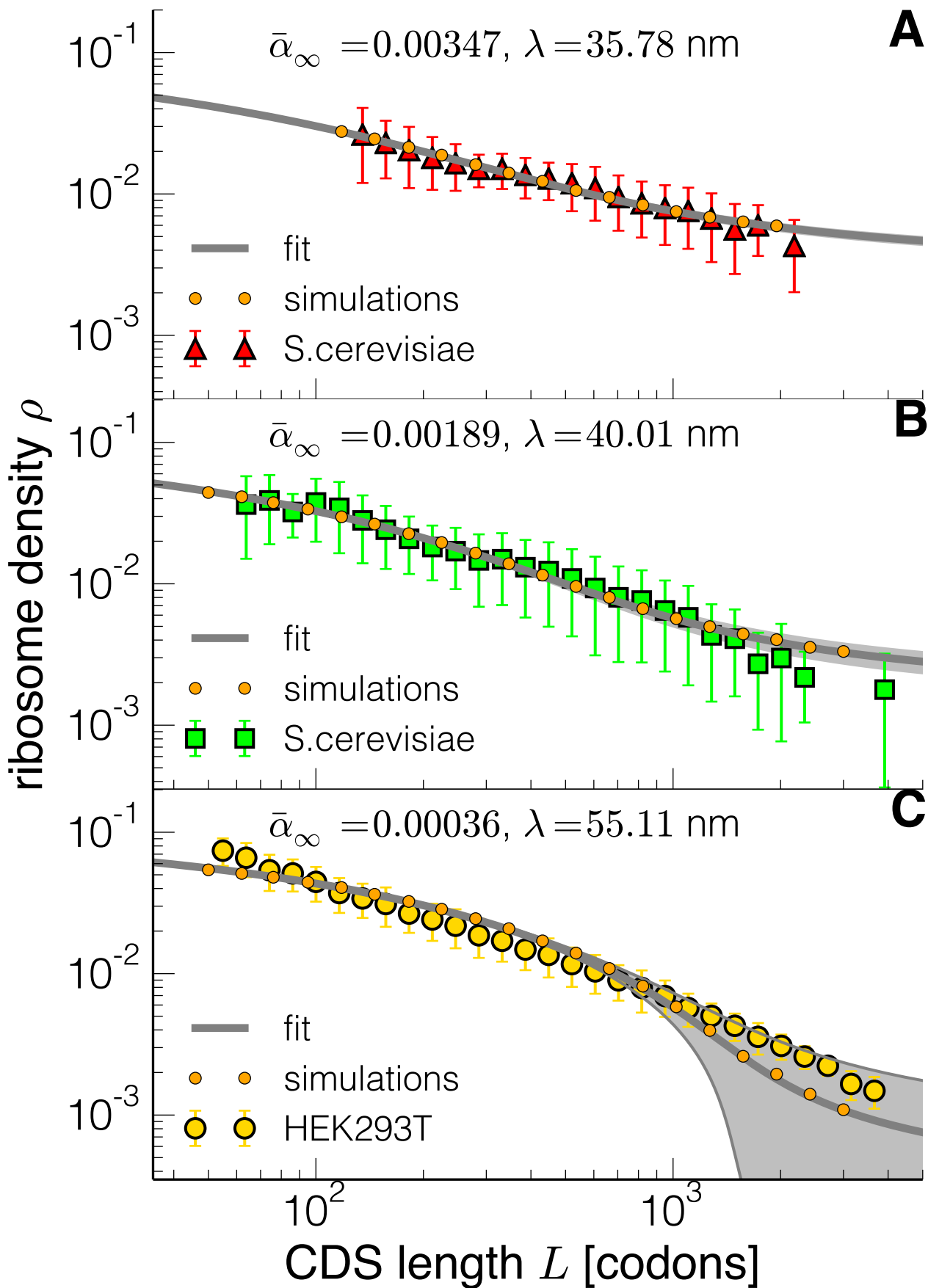

Supplement: Supplementary file 3 — LaTeX Supplementary File [file 41598_2017_17618_MOESM3_ESM.pdf]

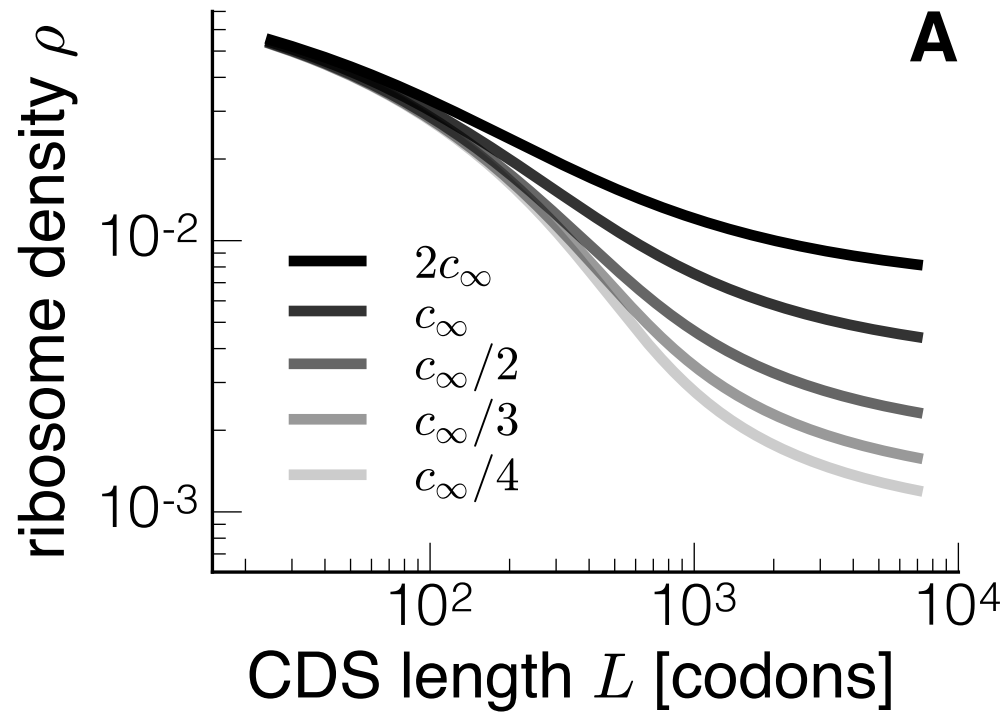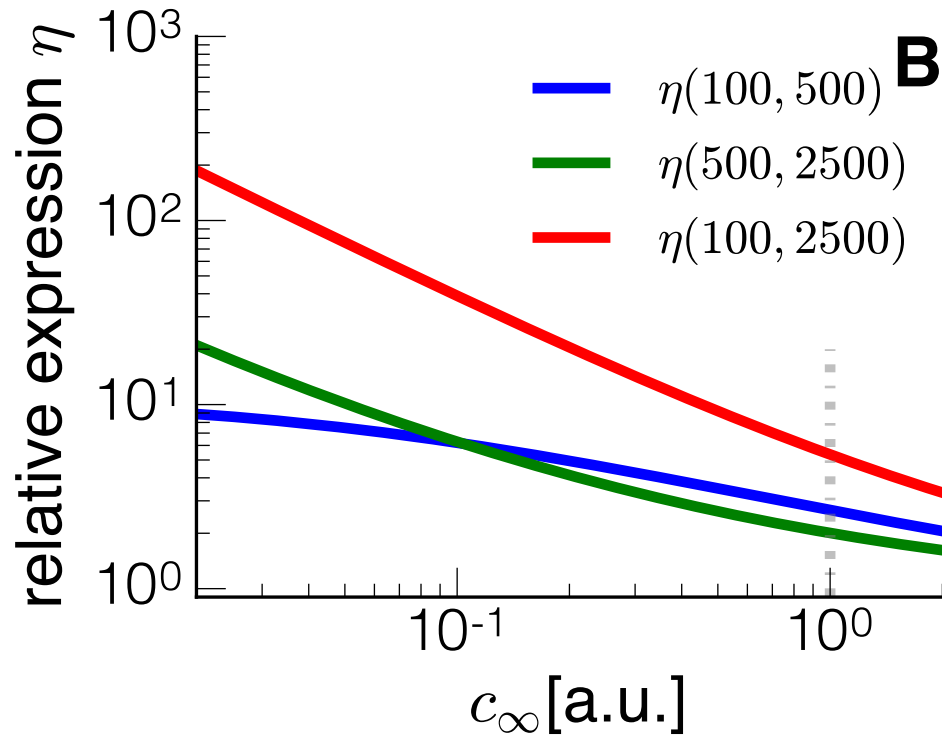

Supplement: Supplementary file 4 — LaTeX Supplementary File [file 41598_2017_17618_MOESM4_ESM.pdf]

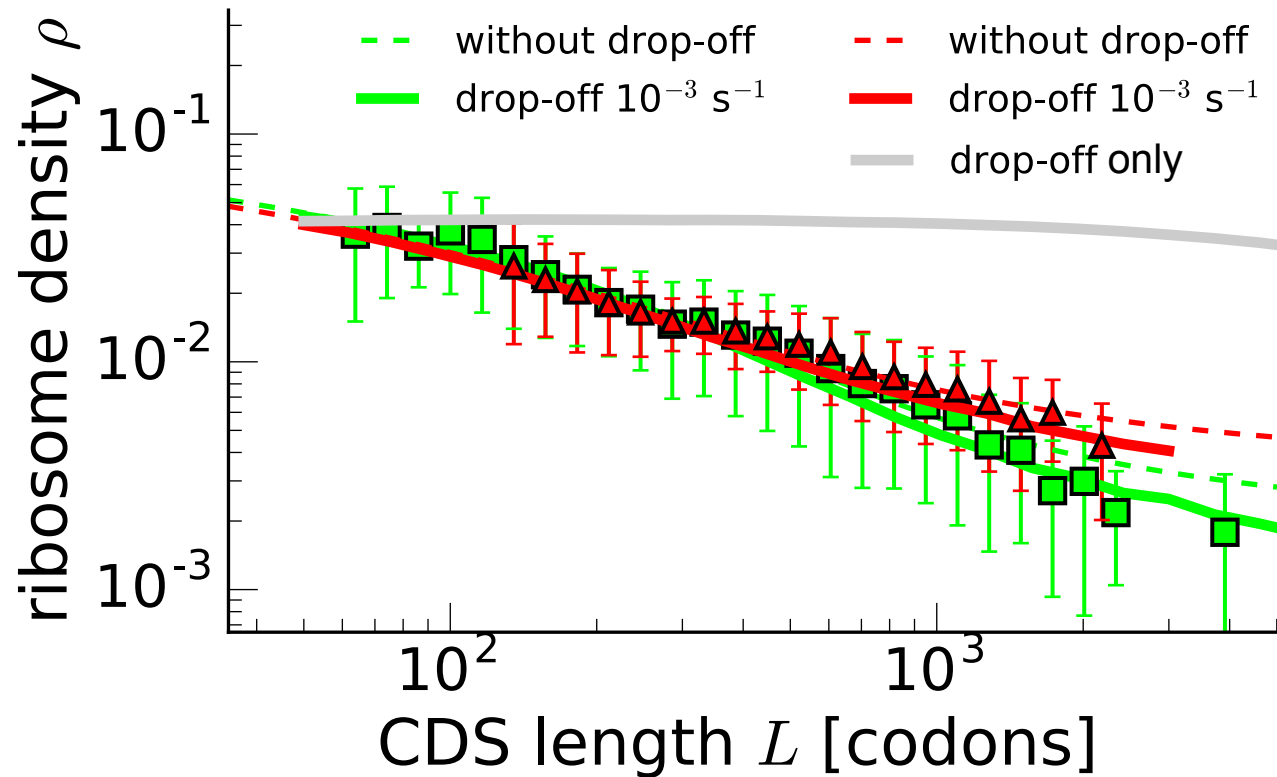

Supplement: Supplementary file 5 — LaTeX Supplementary File [file 41598_2017_17618_MOESM5_ESM.pdf]

open state

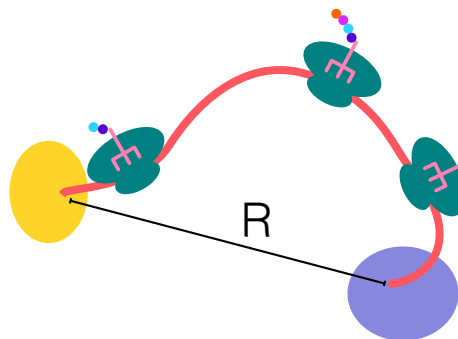

circularised state

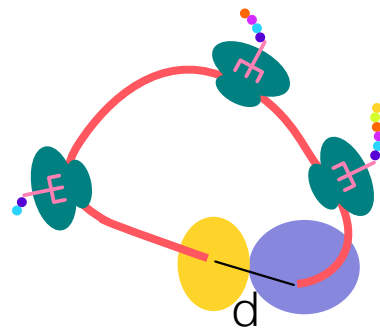

**A**

ribosome density  $\rho$

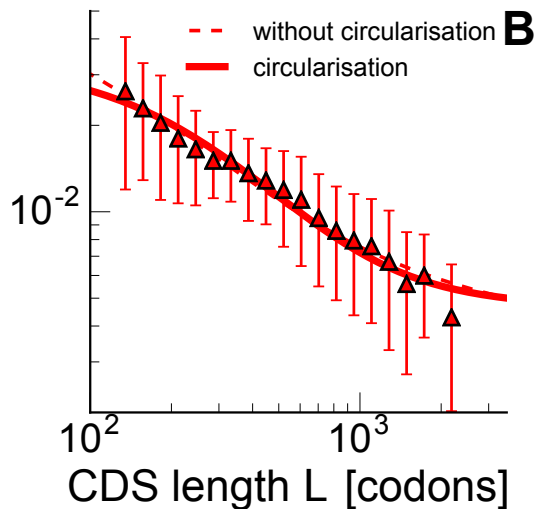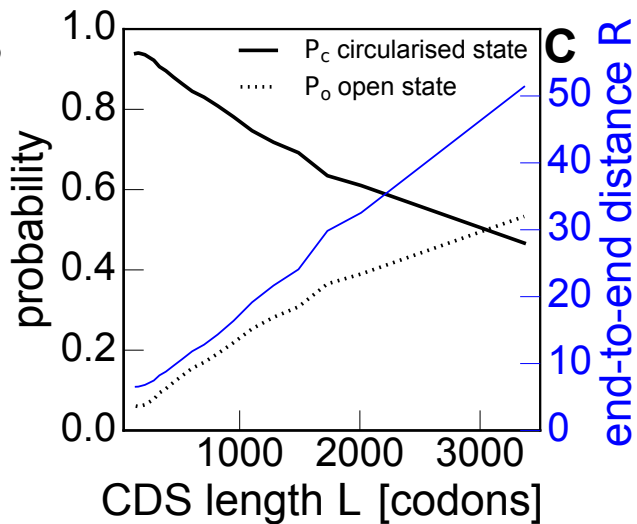

Supplement: Supplementary file 6 — LaTeX Supplementary File [file 41598_2017_17618_MOESM6_ESM.pdf]
