# Supplementary material for: Gene length as a regulator for ribosome recruitment and protein synthesis: theoretical insights
Source: Sci Rep. 2017 Dec 12;7:17409. doi: 10.1038/s41598-017-17618-1 (PMC5727216; doi:10.1038/s41598-017-17618-1)
Supplement: Supplementary file 7 — Supplementary Material [file 41598_2017_17618_MOESM7_ESM.pdf]

Supplementary Material:
Gene length as a regulator for ribosome recruitment and protein synthesis: theoretical insights

Lucas Dias Fernandes[1], **Alessandro de Moura**[2], **and Luca Ciandrini**[3,4,*]

[1]Departamento de Entomologia e Acarologia, Escola Superior de Agricultura ”Luiz de Queiroz” - Universidade de São Paulo, ESALQ - USP, 13418-900, Piracicaba/SP, Brazil
[2]Institute for Complex Systems and Mathematical Biology, University of Aberdeen, Aberdeen, AB24 3UE, UK
[3]DIMNP UMR 5235, Université de Montpellier and CNRS, F-34095, Montpellier, France
[4]Laboratoire Charles Coulomb UMR5221, Université de Montpellier and CNRS, F-34095, Montpellier, France
[*]luca.ciandrini@umontpellier.fr

Contents

1 The Exclusion Process **1**
1.1 The ℓ-TASEP 2
2 Calculation of the background and the feedback concentrations c_∞ and δ_R **4**
2.1 Derivation - Reaction rate 5
2.2 Derivation - Capture probability by a target 5
2.3 Two diffusing subunits 6
3 Details of the mRNA circularisation model **6**
4 Parameter estimation **9**
4.1 Parameters with mRNA circularisation 9
5 Monosome/polysome ratio **9**
References **10**

1 The Exclusion Process

The first mathematical models for translation were devised in the 60's[1,2], incorporating several aspects of non-equilibrium statistical physics to describe the flow of ribosomes on mRNA transcripts. This inspired mathematical approaches for studying the stochastic dynamics of particles in a 1D-chain, from which the *Totally Asymmetric Simple Exclusion Process* (TASEP) emerged[3].

An Exclusion Process consists of a driven gas of moving particles on a discrete chain. Particles hop right or left with given rates (provided the neighbouring site is empty). It uses the simplest possible interaction between particles, which is hard-core exclusion (hence, simple exclusion). The particular case in which the hopping is strictly unidirectional (hence, totally asymmetric) constitutes the TASEP, which it is considered as a minimal model describing transport systems.

Many of the applications to transport systems derive from the formulation of the TASEP in open chains. The model is constructed on a one-dimensional discrete lattice with L sites, where each site can be either empty or occupied by a particle. The occupancy for each site can be defined as n_i, assuming values 0 (empty) or 1 (occupied). Particles hop to the neighbouring site (following unidirectional motion), if it is empty, with a rate p. As the boundaries are open, particles enter the first site of the lattice (if empty) with an entry rate α and leave the lattice with an exit rate β on the last site. Figure 2A of the main text shows a representation of the TASEP.

Once the steady state for this system has been reached, it is possible to define the average occupation for each site, $\rho_i := \langle n_i \rangle$, and the average particle currents J_i, which are defined by the number of particles passing through site i per unit of time. As time and ensemble averages are equivalent for this system, we shall omit this distinction when dealing with the brackets $\langle \cdot \rangle$.

The steady-state solutions for the mean particle density, $\rho := \frac{1}{L}\sum_i \rho_i$, and the average current, $J := \frac{1}{L}\sum_i J_i$, can be obtained in a mean-field approximation (for which spatial correlations are neglected) in the thermodynamic limit ($L \to \infty$). In this work we use this approximation, which turns out to be sufficient for our application (cf. for instance orange circles -simulations,

exact- and grey line -analytical approximation- of the model in Figure 3 of the main text). Full demonstration and more details can be found, for instance, in[4,5]; here we present only the main results.

The phase diagram for the steady state is characterised by three distinct phases, with the boundaries defined by the values of the parameters α and β. For all of these phases, the current is given by the relation $J = p\rho(1-\rho)$. These phases are the low density (LD) phase, the high density phase (HD) and the maximal current (MC) phase, which are described below.

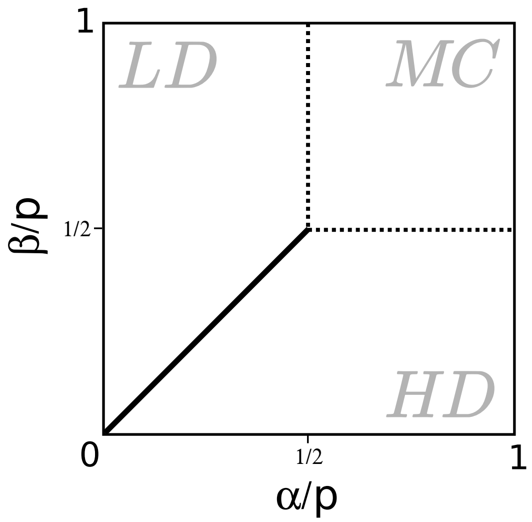

Figure S1. Phase diagram for the TASEP showing three characteristic phases, low density (LD) phase, high density (HD) phase and maximal current (MC) phase, in the $\{\alpha/p, \beta/p\}$-plane, where α, β and p are the entry, exit and hopping rates, respectively. Dotted lines represent continuous second order transitions (between LD and MC phases and between HD and MC phases) on the densities, while bold line represents discontinuous transition on the density (between LD and HD phases).

Low density (LD) - $\alpha < \beta$ and $\alpha/p < 1/2$ - With α small, particles' entry constitutes the limiting step, reaching the steady state as a sparsely occupied lattice, with a mean density of particles $\rho = \alpha/p$. The current is given by $J = \alpha(1-\alpha/p)$.

High density (HD) - $\beta < \alpha$ and $\beta/p < 1/2$ - In this phase the exit rate β is limiting. This generates queues on the lattice, with a mean density $\rho = 1-\beta/p$. For the current, $J = \beta(1-\beta/p)$.

Maximal current (MC) $\alpha/p, \beta/p \geq 1/2$ - With both α and β large, neither entry or exit of particles are limiting steps and a maximal current, limited by the hopping rate p, is reached. In this phase, $\rho = 1/2$ and $J = p/4$.

Transitions between the low density (LD) and the maximal current (MC) phases or between the high density (HD) and the maximal current (MC) phases are continuous in the density, whereas the transition between LD and HD phases is discontinuous in the density. This discontinuous transition line ($\alpha = \beta$ and $\alpha, \beta < p/2$) is characterised by a coexistence of low density and high density phases, as the lattice partitions into regions of the LD density and the HD density with a well-defined boundary between both. This boundary is called a shock, and this scenario, for parameters on the LD-HD boundary line, is also known as shock phase[6].

The phase diagram for the mean field solutions of the steady state in the TASEP model is shown in figure S1.

1.1 The ℓ-TASEP

Many extensions and modifications have been made to the initial framework of the TASEP. Of particular importance to the study of translation is the one commonly referred to as ℓ-TASEP, in which each particle occupies ℓ sites in the chain[7]. In a direct comparison with mRNA translation, each particle then represents a ribosome of size ℓ (measured in units of codon length), reading an mRNA chain of size L, where each site corresponds to one codon. In this model, ribosomes enter the chain with an initiation rate α, and are translocated to the next codon with a homogeneous rate p. At the end of the chain, they are terminated with a rate β.

After a transient time, this system reaches a steady state, in which the average ribosomal density, $\rho = N/L$ (where N is the average number of ribosomes at steady state and L the size of the transcript), and the average ribosomal current, J (number of particles passing through each site per unit of time), fluctuate around stationary values.

In[7] the authors obtained analytical expressions for the ribosomal density and the ribosomal current for the system with open boundaries. They also performed Monte Carlo simulations that showed good agreement with the analytical solutions obtained in the approximation.

The phase diagram obtained for the ℓ-TASEP is qualitatively analogous to the one obtained for the standard TASEP, but with quantitative corrections on the phase boundaries and on the stationary values of density and current, due to the size ℓ of the particles. The relation that Shaw et al.[7] obtained between ribosomal current and density is given by

$$J = \frac{p\rho(1-\rho\ell)}{1-\rho(\ell-1)}$$

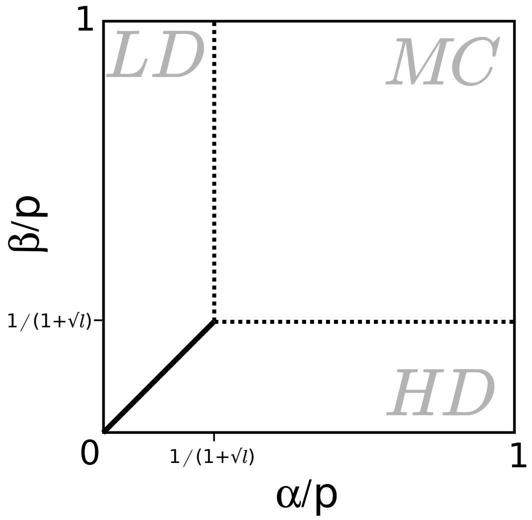

Figure S2. Phase diagrams for the ℓ-TASEP model showing the same three characteristic phases, low density (LD) phase, high density (HD) phase and maximal current (MC) phase, in the $\{\alpha/p, \beta/p\}$-plane, but with corrections in the values of density and current for each phase (see equations S1 to S3). Boundaries of the phases are also corrected by a factor $2/(1+\sqrt{\ell})$. Dotted lines again represent continuous transitions on the densities, while bold line represents discontinuous transition on the density.

Thus, taking the corrections in the density into account[7], the mean-field values and description of the phases for the ℓ-TASEP are given by:

Low density (LD) - $\alpha < \beta$ and $\alpha/p < \dfrac{1}{1+\sqrt{\ell}}$;

$$\rho = \frac{\left(\frac{\alpha}{p}\right)}{1+\left(\frac{\alpha}{p}\right)(\ell-1)}, \qquad J = \frac{\alpha\left(1-\left(\frac{\alpha}{p}\right)\right)}{1+\left(\frac{\alpha}{p}\right)(\ell-1)} \tag{S1}$$

High density (HD) - $\beta < \alpha$ and $\beta/p < \dfrac{1}{1+\sqrt{\ell}}$;

$$\rho = \frac{1}{\ell}\left(1-\frac{\beta}{p}\right), \qquad J = \frac{\beta\left(1-\left(\frac{\beta}{p}\right)\right)}{1+\left(\frac{\beta}{p}\right)(\ell-1)} \tag{S2}$$

Maximal current (MC) $\alpha/p, \beta/p \geq \dfrac{1}{1+\sqrt{\ell}}$;

$$\rho = \frac{1}{\sqrt{\ell}(1+\sqrt{\ell})}, \qquad J = \frac{p}{(1+\sqrt{\ell})^2} \tag{S3}$$

From these relations with $\ell = 1$ we recover the expressions for densities and currents of the standard TASEP. Figure S2 shows the phase diagram for the ℓ-TASEP.

We emphasise that the density always depends on the ratios $\bar{\alpha} = \alpha/p$ and $\bar{\beta} = \beta/p$. In the main text we use

$$J(\bar{\alpha}) \equiv J(\alpha, p)/p = \frac{\bar{\alpha}(1-\bar{\alpha})}{1+\bar{\alpha}(\ell-1)}.$$

By considering $\ell = 10$, we obtain a critical value of $\bar{\alpha} = 1/(1+\sqrt{\ell}) \sim 0.24$, which plugged into S1 provides a value of the particle density of ~ 0.076.

2 Calculation of the background and the feedback concentrations c_∞ and δ_R

We consider the initiation rate α of mRNA translation as a sum of two terms:

$$\alpha = \alpha_\infty + \gamma = \alpha_0(c_\infty + \delta_R), \tag{S4}$$

where α_∞ refers to the rate of initiation of ribosomes that belongs to the cytoplasmatic pool, and γ to the rate of recycling, or the rate with which ribosomes in the termination stage of the translation of a given transcript are reinserted at the 5' end of the mRNA to begin the process again. Alternatively, one can imagine that the initiation reaction occurs with the product between an affinity α_0 and the local concentration of ribosomes in the reaction volume surrounding the ribosome binding site. This concentration is given by c_∞, the background concentration far away from the transcript, plus the contribution δ_R due to the proximity of the 3'end acting as a source of ribosomes. An illustration of the process is shown in figure 2B of the main text. For the sake of simplicity, we consider ribosomes as particles performing free diffusion when they are not bound to the mRNA. To start with, we neglect the separation of ribosomes into its 40S and 60S subunits, and consider them as individual diffusing particles. An extension can be found in Section 2.3 of this Supplementary Material.

The term α_∞ can be obtained if we consider diffusing particles in a finite (cell) volume V that diffuse freely until they find the spherical reaction centre of radius a (the volume of the reaction centre being small compared to V). If there are N such particles, the average time it takes for a particle to reach the reaction centre is given by $\tau_{rc} = 1/4\pi D a c_\infty$ (see Supplementary material, section 2.1, for derivation), where D is the diffusion coefficient of the particles. We may also take into account a probability of reaction κ; this describes the probability that, after reaching the reaction centre, the process is indeed initiated. Essentially, κ represents the affinity of the ribosome for the mRNA, and it serves to rescale the time for the binding reaction. This time can vary from mRNA to mRNA due to properties of the sequence, like secondary structures in the 5'UTR.

If a is the radius of a reaction volume as shown in figure 2B of the main text, then α_∞ can be estimated by:

$$\alpha_\infty = \frac{1}{\tau_{rc}}\kappa = 4\pi D a \kappa c_\infty \equiv \alpha_0 c_\infty, \tag{S5}$$

where $c_\infty = N/V$ is the homogeneous background ribosome concentration in the cytoplasm, and we have included all the other properties in the α_0 term.

The term γ instead describes how the local concentration of ribosomes is affected by the feedback induced by the terminating ribosomes. To obtain this term we first have to consider the probability of a given particle reaching a sphere of radius a (which again we treat as the reaction centre for the initiation process), starting from a distance R from the centre of the sphere (the end-to-end distance of the mRNA). Since in three dimensions diffusion is non-recurrent, there is a chance that the particle escapes to infinity without ever reaching the sphere (here we again consider that the volume V of the cell is much bigger than the sphere volume, so that the approximation of infinite space is still good enough). For dimensionless particles, the probability to end up in the reaction volume is a/R (see Supplementary material, section 2.2, for derivation). Assuming that translation is at steady state, to obtain the rate γ we then need to weight this probability with the current J of the source point (the ribosomal current at the end of the transcript). In other words, the average time it takes for a ribosome to appear at the end of the transcript is $\tau_J = 1/J$. Finally, we also consider the probability κ, as defined before. If we consider, for now, the separation R between the two ends of the transcript to be fixed, with the previous prescriptions, we have for the rate of recycling, γ:

$$\gamma = \frac{a}{R}\kappa\frac{1}{\tau_J} = a\kappa\frac{J}{R}. \tag{S6}$$

Interestingly, this relation for γ induces a feedback between the initiation and the overall process (the current J) or, in other words, the elongation process affects the initiation via the recycling process.

Substituting equations S5 and S6 into equation S4, the total initiation rate α can be written as:

$$\alpha = 4\pi D a \kappa c_\infty + a\kappa \frac{J}{R} = \alpha_0 (c_\infty + \delta_R) \tag{S7}$$

where we use again $\alpha_0 = 4\pi D a\kappa$ and define $\delta_R = \frac{J}{4\pi D R}$; α_0 can then be interpreted as the rate constant of the initiation process (in principle depending on the mRNA and on the diffusion process), and δ_R represents the local change in the concentration around the reaction volume (to be added to the global concentration c_∞).

Defining $\lambda = a\kappa$, equation S7 can be written as:

$$\alpha = 4\pi D a \kappa c_\infty + a\kappa \frac{J}{R} = \alpha_\infty + \lambda \frac{J}{R}\,. \tag{S8}$$

For simplicity, we favour this last expression for α and we will use α_∞ and λ as free parameters of our model. The dimensions of α_∞ and λ are $1/[time]$ and $[length]$, respectively. We shall use s^{-1} as units for α_∞ and nm for λ (assuming 1 codon $\sim$ 1 nm).

2.1 Derivation - Reaction rate

The derivation of the reaction rate, defined as the rate with which diffusing particles reach an absorbing object (here an analogy for a reaction centre), is a well-known result and it is useful in several physical and biological applications. In this subsection we follow the development presented in[8] as a reference.

Calculating the time it takes for a given molecule or particle to find a binding site is equivalent to ask how much time it takes for a particle to reach a given reaction sphere. We assume that the radius a of the sphere is very small compared to the radius of the whole (spherical) volume V in which particles can diffuse. Let us consider that far away from the reaction sphere there is a concentration $c_\infty = N/V$ of particles, which is held constant.

We obtain the steady-state solution by solving the diffusion equation with absorbing boundary conditions on the reaction volume:

$$\nabla^2 c(\vec{r}) = 0 \quad \text{with} \quad c(\vec{r} \in \partial B) = 0,\, c(\vec{r} \to \infty) = c_\infty\,, \tag{S9}$$

where ∂B is the boundary of the sphere of radius a, and $\vec{r}$ is the position vector with the origin at the centre of the reaction volume.

Solution of equation S9 gives:

$$c(r) = c_\infty \left(1 - \frac{a}{r}\right) \tag{S10}$$

From Fick's law, the flux of particles, $\vec{J}$, is given by $\vec{J} = -D\vec{\nabla}c$. The rate, K, with which particles are absorbed into the small sphere is then given by:

$$K = \int_{\partial B} \vec{J} \cdot d\vec{\sigma} = -D \int_{\partial B} \vec{\nabla} c \cdot d\vec{\sigma} = -4\pi D a^2 \left(\frac{dc}{dr}\right)_{r=a}\,.$$

From S10 we can obtain $\left(\frac{dc}{dr}\right)_{r=a} = \frac{c_\infty}{a}$. Thus, we have:

$$K = -4\pi a D c_\infty\,.$$

As can be seen, K has the correct unit of rate (1/[time]). Hence, the average time it takes for a particle to reach the reaction sphere is given by:

$$\tau_{rc} = 1/|K| = 1/4\pi a D c_\infty\,.$$

2.2 Derivation - Capture probability by a target

The capture probability of a diffusing particle by an absorbing object can be obtained as an approximation of the exit probability, which is related to the problem of a bounded particle reaching a given portion of the boundary without ever reaching the other portion. This is also a known result and again we follow[8].

Let us consider a random walk in one dimension on the interval $[0,N]$. Let ε_n be the probability that the walk reaches site N without visiting site 0 starting at site n. This probability satisfies the recurrence equation:

$$\varepsilon_n = \frac{1}{2}(\varepsilon_{n-1} + \varepsilon_{n+1}). \tag{S11}$$

In fact, a random walker starting from site n can either step left or right with equal probability $1/2$. Then the probability of reaching N without passing via the site 0, can be written as in equation S11 in terms of ε_{n-1} and ε_{n+1}. Boundary conditions are given by $\varepsilon_0 = 0$ (if the walker is already at site 0, the probability of reaching N without visiting site 0 is zero) and $\varepsilon_N = 1$ (for a similar reasoning).

Generalizing the reasoning that led to equation S11 for an arbitrary domain, the exit probability is given in the continuum limit by the Laplace equation subject to the following boundary conditions:

$$\nabla^2 \varepsilon(\vec{r}) = 0 \quad \text{with} \quad \varepsilon(\vec{r} \in \partial B_-) = 0, \varepsilon(\vec{r} \in \partial B_+) = 1\,, \tag{S12}$$

where the boundary ∂B of the arbitrary domain was partitioned into two disjoint subsets ∂B_- and ∂B_+ (∂B=$\partial B_- \cup \partial B_+$). Thus, we ask for the probability $\varepsilon(\vec{r})$ that the particle reaches the domain via ∂B_+ without visiting ∂B_-.

To be more concrete, consider the problem of a particle in a 3D space, bound by two concentric spheres: an internal, with radius a, and an external, with radius a_∞. The vector $\vec{r}$ is the position of the particle with its origin at the centre of the sphere of radius a. If the particle starts at a distance $r = R$ from the centre of the reaction volume sphere with $R \in (a, a_\infty)$, it is then possible to compute the probability that it will hit this sphere (reaction volume) before reaching the external sphere (leaving to the infinity). This will be obtained by solving equation S12, subject to the boundary conditions $\varepsilon(a_\infty) = 0$ and $\varepsilon(a) = 1$. The solution is given by:

$$\varepsilon(R) = \frac{(a/R) - (a/a_\infty)}{1 - (a/a_\infty)}. \tag{S13}$$

With the solution given by equation S13, taking the limit $a/a_\infty \to 0$ we obtain:

$$\varepsilon(R) = \frac{a}{R}. \tag{S14}$$

2.3 Two diffusing subunits

In this section we develop a coarse-grained argument to consider the concurrent diffusion of the two ribosomal subunits. Initiation occurs if both small *and* large subunits are present. This would lead to the product of two terms of the type shown in Eq.(S14), leading to a dependence $\sim 1/R^2$. Then the expression for the initiation rate found in Eq.(S8) would change to:

$$\alpha = \alpha_\infty + \lambda_2 \frac{J}{R^2}\,, \tag{S15}$$

where λ_2 has dimensions of $length^2$. The quantitative results do not change significantly and are shown in the Figures S9 and S12.

3 Details of the mRNA circularisation model

If G_c and G_o are the free energies for the *circularised* and *open* states of the transcript, respectively (see Figure 5 of the main text), then the equilibrium probability for the occurrence of the circularised state is given by

$$P_c = \frac{e^{-G_c/(k_B T)}}{e^{-G_c/(k_B T)} + e^{-G_o/(k_B T)}} = \frac{1}{1 + e^{\Delta G/(k_B T)}}. \tag{S16}$$

The free energies should be computed as it follows:

$$\begin{aligned} G_c - G_o &= (E_c - TS_c) - (E_o - TS_o) \\ &= \Delta E - T\Delta S\,, \end{aligned}$$

where the energy of the circularised state E_c takes into account the bending energy of the mRNA molecule as well as the interaction energy $\varepsilon k_B T$ between the two ends, mediated by the PABP and the 5'-cap. We also consider the bending energy for

the open state very small when compared to the circularised one, so that we set $E_o \approx 0$. Thus, we have (referring for instance to Phillips *et al.*[9]):

$$\Delta E = E_c - E_o = \left(\frac{2\pi^2 l_p}{L} + \varepsilon\right) k_B T\,, \tag{S17}$$

where L is the length of the mRNA molecule, l_p is the persistance length and $\varepsilon < 0$.

Similarly to the procedure used to obtain Eq. (S14), we obtain the probability of having the two ends separated by a distance d in a three dimensional random walk:

$$p_{crw} = \left(\frac{6}{\pi N^3}\right)^{1/2} \left(\frac{d}{a}\right)^3,$$

where a is the Kuhn length and N is the number of Kuhn segments. But since $Na = L$ and $a = 2l_p$, we have:

$$\begin{aligned} p_{crw} &= d^3 \sqrt{\frac{6}{\pi}} \frac{\sqrt{N^3}}{N^3 a^3} = \left(\frac{d}{L}\right)^3 \sqrt{\frac{3}{4\pi}\left(\frac{L}{l_p}\right)^3} \\ &= \left(\frac{d}{\sqrt{l_p L}}\right)^3 \sqrt{\frac{3}{4\pi}}\,. \end{aligned}$$

We will identify this state with the two ends separated by d as the circularised state (see also Figure 5 and the main text).

Then we have for the entropies of circularised and open states:

$$\begin{aligned} \Delta S = S_c - S_o &= k_B[\ln(p_{crw}) - \ln(1 - p_{crw})] \\ &= k_B \ln\left(\frac{p_{crw}}{1 - p_{crw}}\right) \\ &= k_B \ln\left(\frac{1}{\frac{1}{p_{crw}} - 1}\right) \\ &= k_B \ln\left[\frac{1}{\left(\frac{\sqrt{l_p L}}{d}\right)^3 \sqrt{\frac{4\pi}{3}} - 1}\right] \\ &= -k_B \ln\left[\left(\frac{\sqrt{l_p L}}{d}\right)^3 \sqrt{\frac{4\pi}{3}} - 1\right] \end{aligned} \tag{S18}$$

Thus, with Eqs. (S17) and (S18), we have:

$$\begin{aligned} \Delta G &= \Delta E - T\Delta S \\ &= \left(\frac{2\pi^2 l_p}{L} + \varepsilon\right) k_B T + k_B T \ln\left[\left(\frac{\sqrt{l_p L}}{d}\right)^3 \sqrt{\frac{4\pi}{3}} - 1\right] \\ &= k_B T \left\{\left(\frac{2\pi^2 l_p}{L} + \varepsilon\right) + \ln\left[\left(\frac{\sqrt{l_p L}}{d}\right)^3 \sqrt{\frac{4\pi}{3}} - 1\right]\right\} \end{aligned} \tag{S19}$$

which we can plug in Eq. (S16) to obtain the equilibrium probability P_c of the circularised state:

$$\begin{aligned} P_c &= \frac{1}{1 + e^{\Delta G/(k_B T)}} \\ &= \frac{1}{1 + \left[\left(\frac{\sqrt{l_p L}}{d}\right)^3 \sqrt{\frac{4\pi}{3}} - 1\right] e^{\left(\frac{2\pi^2 l_p}{L} + \varepsilon\right)}}\,. \end{aligned} \tag{S20}$$

We notice that in Eq.(S20) one should replace the persistence length l_p with the effective persistence length l_{eff} in order to consider the stiffness effect induced by the ribosomes (see Eq. (2) and the main text). We are then able to examine how the free energy ΔG behaves by changing the physical properties of the mRNA, for instance its length or its effective persistence length via the ribosome density ρ (Figure S3). As expected, ΔG grows by increasing both the coding sequence length and the ribosome density ρ, meaning that the probability P_c decreases according to Eq. (S16). We also notice that, for a fixed relatively high value of the density ρ (we remind that the maximal value of the density in the LD is ~ 0.076), $\Delta G(L)$ has a minimum for small values of L (Figure S3A).

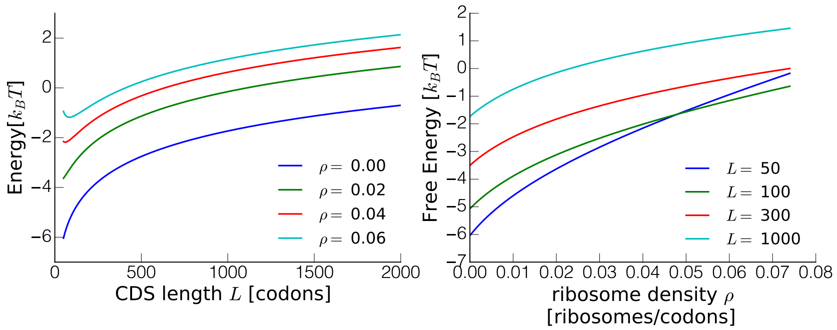

Figure S3. The free energy difference ΔG between the circularised and open state as a function of L for different values of ρ (**A**), and of ρ for different values of L (**B**), see Eq. (S19)).

With this extension to the model, taking circularisation into account, we are able to obtain plausible fitting parameters for the Mackay (see Figure 5 of the main text) and Arava datasets (see Figure S11A). The fitting parameters can be found in the following section. Attempts to fit the Hendrickson (HEK293T) dataset yields positive values for ε for the best fit, alongside the same values we have obtained before for α_∞ and λ in the model without circularisation. Nevertheless, plausible values for the parameters can still be attempted considering circularisation in the Hendrickson dataset. Even though these parameterss do not correspond to best fits, according to the fitting criteria of the intrinsic functions used in the software Mathematica, the results are still able to describe the basic phenomenology of the length-dependence in the experimental values of the density. The results for attempted parameters in the Hendrickson dataset can be seen in Figure S11B.

4 Parameter estimation

The parameters have been estimated for the three datasets used in the main text (see Fig.3) by fitting the numerical solutions as explained in the Materials and Methods section. Below we summarise the parameter with their standard error (α_∞ in s^{-1}, λ and λ_2 in nm and ε in $k_B T$ units).

Dataset	Parameters	Estimate	Standard Error	P-value
Mackay (yeast)	$\bar{\alpha}_\infty$	0.0035	0.0003	$< 10^{-10}$
	λ	35.8	0.8	$< 10^{-19}$
Arava (yeast)	$\bar{\alpha}_\infty$	0.0019	0.0004	$< 10^{-4}$
	λ	40.0	0.9	$< 10^{-23}$
Hendrickson (HEK293T)	$\bar{\alpha}_\infty$	0.0004	0.0005	0.50
	λ	40.0	0.9	$< 10^{-19}$

Table S1. Values of the parameters $\bar{\alpha}_\infty$ and λ estimated for the three different datasets used in Fig.3

Dataset	Parameters	Estimate	Standard Error	P-value
Mackay (yeast)	$\bar{\alpha}_\infty$	0.0059	0.0004	$< 10^{-10}$
	λ_2	1040	50	$< 10^{-13}$
Arava (yeast)	$\bar{\alpha}_\infty$	0.0047	0.0007	$< 10^{-5}$
	λ_2	1070	50	$< 10^{-15}$
Hendrickson (HEK293T)	$\bar{\alpha}_\infty$	0.0021	0.0007	0.0036
	λ_2	1860	80	$< 10^{-19}$

Table S2. Values of the parameters $\bar{\alpha}_\infty$ and λ estimated for the three different datasets used in Fig. S9 with the diffusion of two ribosomal subunits.

4.1 Parameters with mRNA circularisation

Dataset	Parameters	Estimate	Standard Error	P-value
Mackay (yeast)	$\bar{\alpha}_\infty$	0.0047	0.0006	$< 10^{-6}$
	λ_2	7	0.6	$< 10^{-9}$
	ε	-8.3	0.4	$< 10^{-13}$
Arava (yeast)	$\bar{\alpha}_\infty$	0.0024	0.0008	$< 10^{-3}$
	λ_2	32	11	0.010
	ε	-4.5	1.9	0.025

Table S3. Values of the parameters $\bar{\alpha}_\infty$ and λ estimated for the three different datasets used in Fig. 5 and Figure S11A for the mRNA circularisation model.

5 Monosome/polysome ratio

In our stochastic simulations the amount of ribosomes on a mRNA fluctuates around a mean value $\bar{n} = \rho L$. However, there is a non-negligible probability to find, during the time of a simulation, a transcript with $n \neq \bar{n}$ ribosomes. We followed the time evolution of the system and computed the probability of observing $n = 1$ translating ribosomes, and $n > 1$ ribosomes, from which we can obtain the monosome/polysome ratio from the simulations.

By increasing the CDS length we observe a reduction of the monosome/polysome ratio following a power-law behaviour. These results seem to qualitatively correspond to the measurements of a recent experimental work[10].

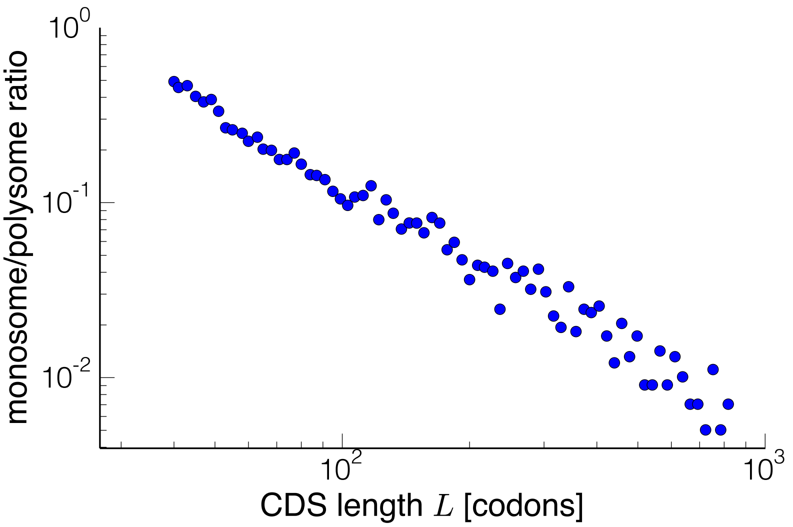

Figure S4. Ratio between actively translating monosomes and polysomes as a function of the CDS length obtained by simulating the model with the parameters $p = 10.0\ s^{-1}$, $\alpha_\infty = 0.036\ s^{-1}$ and $\lambda = 36$ codons.

References

1. MacDonald, C. T., Gibbs, J. H. & Pipkin, A. C. Kinetics of biopolymerization on nucleic acid templates. *Biopolymers* **6**, 1–25 (1968). DOI 10.1002/bip.1968.360060102.

2. MacDonald, C. T. & Gibbs, J. H. Concerning the kinetics of polypeptide synthesis on polyribosomes. *Biopolymers* **7**, 707–725 (1969).

3. Spitzer, F. Interaction of Markov processes. *Advances in Mathematics* **5**, 246–290 (1970).

4. Derrida, B., Domany, E. & Mukamel, D. An exact solution of a one-dimensional asymmetric exclusion model with open boundaries. *Journal of Statistical Physics* **69**, 667–687 (1992).

5. Blythe, R. A. & Evans, M. R. Nonequilibrium steady states of matrix-product form: a solver's guide. *Journal of Physics A: Mathematical and Theoretical* **40**, R333 (2007).

6. Derrida, B. An exactly soluble non-equilibrium system: the asymmetric simple exclusion process. *Physics Reports* **301**, 65–83 (1998).

7. Shaw, L. B., Zia, R. K. P. & Lee, K. H. Totally asymmetric exclusion process with extended objects: A model for protein synthesis. *Phys. Rev. E* **68**, 021910 (2003). DOI 10.1103/PhysRevE.68.021910.

8. Krapivsky, P. L., Redner, S. & Ben-Naim, E. *A Kinetic View of Statistical Physics* (Cambridge University Press, 2010).

9. Phillips, R., Kondev, J., Theriot, J. & Orme, N. *Physical Biology of the Cell* (Garland Science, 2013).

10. Heyer, E. E. & Moore, M. J. Redefining the Translational Status of 80s Monosomes. *Cell* **164**, 757–769 (2016). DOI 10.1016/j.cell.2016.01.003.

11. Lorenz, R. *et al.* Vienna rna package 2.0. *Algorithms for Molecular Biology : AMB* **6**, 26–26 (2011). DOI 10.1186/1748-7188-6-26.

12. Nagalakshmi, U. *et al.* The transcriptional landscape of the yeast genome defined by rna sequencing. *Science* **320**, 1344–1349 (2008). DOI 10.1126/science.1158441.

13. Ringnér, M. & Krogh, M. Folding free energies of 5'-utrs impact post-transcriptional regulation on a genomic scale in yeast. *PLOS Computational Biology* **1**, 1–8 (2005). DOI 10.1371/journal.pcbi.0010072.

14. Ciandrini, L., Stansfield, I. & Romano, M. C. Ribosome traffic on mRNAs maps to gene ontology: genome-wide quantification of translation initiation rates and polysome size regulation. *PLoS Comput. Biol.* **9**, e1002866 (2013). DOI 10.1371/journal.pcbi.1002866.

15. Lanza, A. M., Curran, K. A., Rey, L. G. & Alper, H. S. A condition-specific codon optimization approach for improved heterologous gene expression in saccharomyces cerevisiae. *BMC Systems Biology* **8**, 33 (2014). DOI 10.1186/1752-0509-8-33.

Supplementary Figure S5

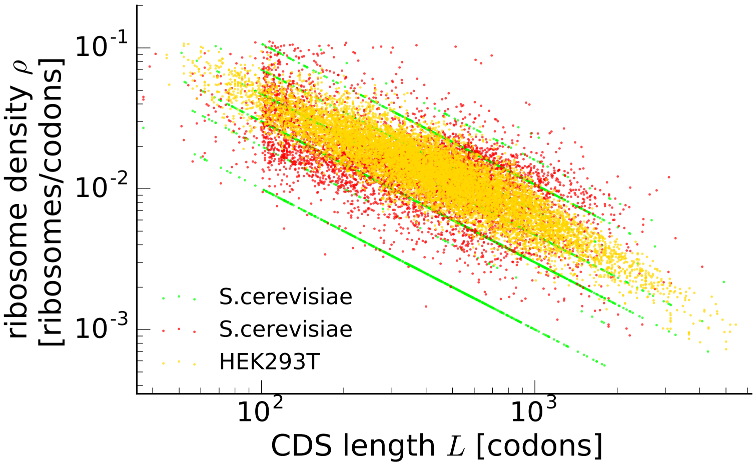

Figure S5. Scatter plots (each point representing a gene) of the experimental ribosomal densities for the three datasets used in this work (see Figure 1 of the main text for the colour code). The length dependence is evident even in the scatter plots and it is not an effect of the binning averages performed in Figure 1. The green points seem discretised because of the resolution of the densities provided in the corresponding dataset (Arava).

Supplementary Figure S6

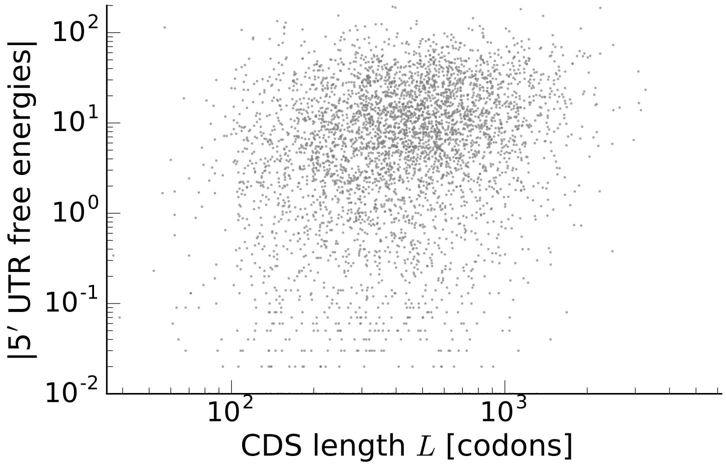

Figure S6. Scatter plot of the absolute value of the free energy strength of secondary structures in the 5'UTRs (yeast) *vs* the CDS length L. For each point we calculated the 5'UTR free energy computed with RNAfold Vienna package[11] for the 5'UTR sequences taken from Nagalakshmi *et al.*[12]. The weak correlation (Pearson $r = 0.147$, p-value $< 10^{-21}$) seems to suggest that the mRNA dependent tuning of the ribosome binding affinities cannot alone produce the length dependent translation efficiency. More rigorous analyses are prone to indicate an even smaller correlation (Pearson $r = 0.01$, p-value 0.5) even with ribosome densities (Pearson $r = 0.09$, p-value $< 10^{-10}$)[13]

Supplementary Figure S7

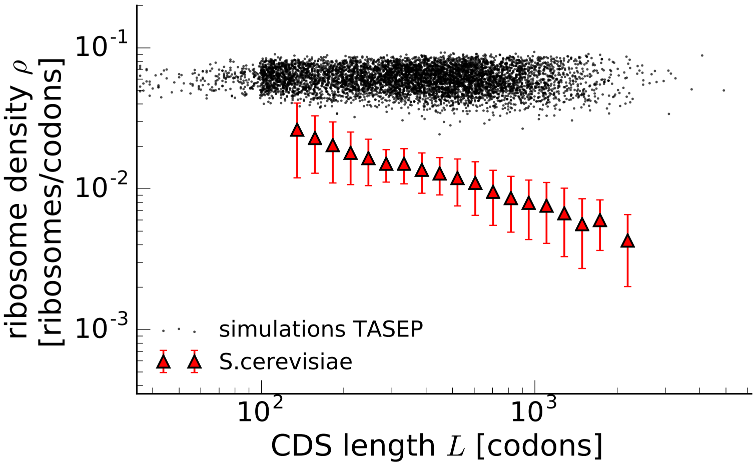

Figure S7. Geneome-wide simulations of a standard TASEP with codon-dependent rates ($\ell = 10$). In these simulations the initiation rate α is fixed to be large in order to *isolate the effects of elongation*. If elongation rates were, even in part, responsible of the length-dependent behaviour of the density, in this figure we would have observed ρ decreasing with L. Each black dot in the figure represent the outcome of the simulation of a gene; as a reference, we plot the experimental densities of the Mackay dataset (see Figure 1 of the main text).

Supplementary Figure S8

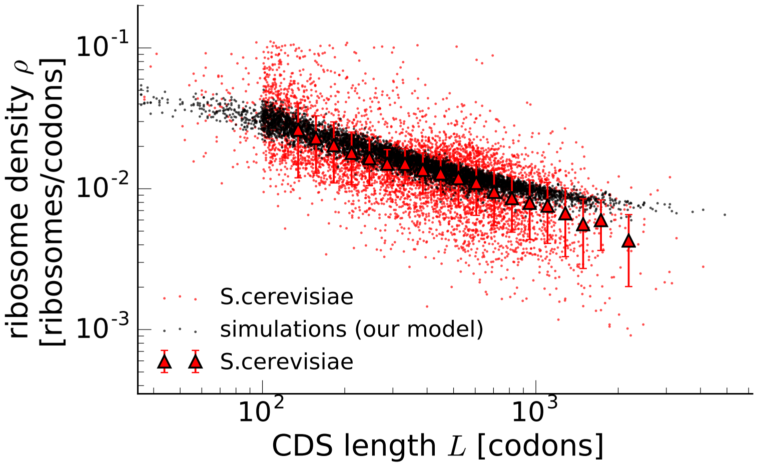

Figure S8. Genome-wide simulations of our translation model with feedback, equivalent of Figure 3 of the main text with codon-dependent hopping rates. Red dots are experimental ribosome densities and red triangles the binned averages (Mackay dataset, cf. Figures 1 and S5); black dots are simulated outcomes of the model with hopping rates from Ciandrini *et al.*[14]. Codon-dependent rates are responsible for the spread in the black cloud, and we attribute the differences with the experimental data to variations in the mRNA-dependent binding affinities, not considered in this work since we used averaged values (see main text).

Supplementary Figure S9

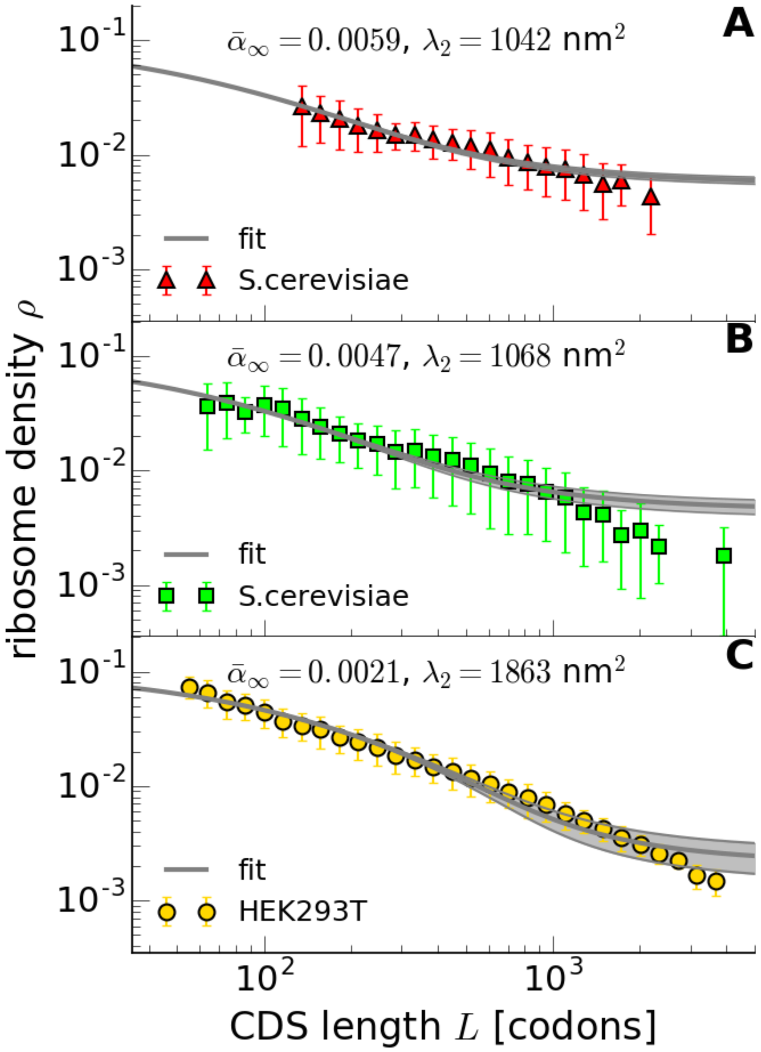

Figure S9. Equivalent to Fig.3, considering the diffusion of the two subunits as explained in Eq.(S15). The standard errors and p-values are summarised in table S3.

Supplementary Figure S10

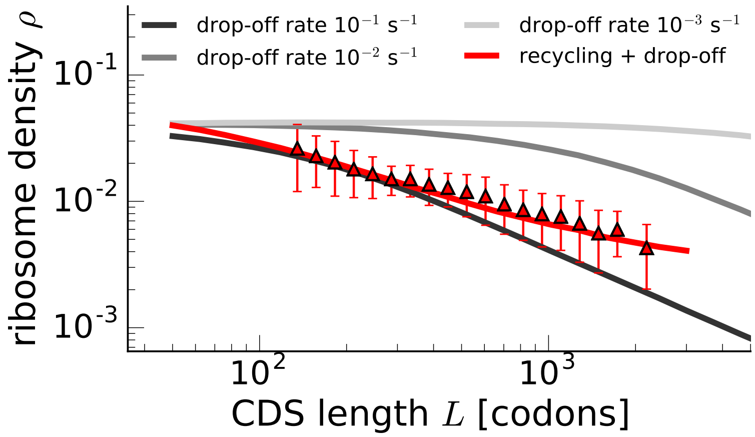

Figure S10. In this figure we simulated a standard exclusion process with $p = 10$ s^{-1} and $\ell = 10$ codons. The initiation rate is fixed to match the density of short transcripts ($\alpha = 0.7$ s^{-1}). In red we report the model with feedback and drop-off as presented in the main text for the Mackay dataset (red triangles). For the typical value of drop-off rate $\delta = 10^{-3}$ s^{-1} we do not observe a length dependence on the density ρ, which leads us to conclude that ribosome drop-off cannot be the only responsible for such a behaviour. Instead, to notice a dependence of the density on the length one should push δ to unrealistic values ($\delta = 10^{-1}$ s^{-1} in the figure).

Supplementary Figure S11

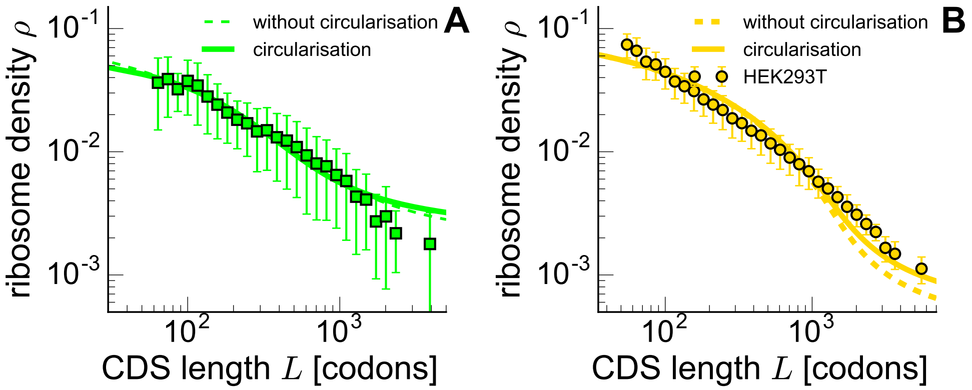

Figure S11. In this figure we show the fit of the model that includes mRNA circularisation for the Arava dataset (**A**) and the Hendrickson dataset (**B**). The estimated parameters for (**A**) are given in section 4. The parameters used to plot the example in panel (**B**) are: $\bar{\alpha}_{\infty} = 0.0005$, $\lambda = 55$ nm, $\varepsilon = -1\,k_b T$.

Supplementary Figure S12

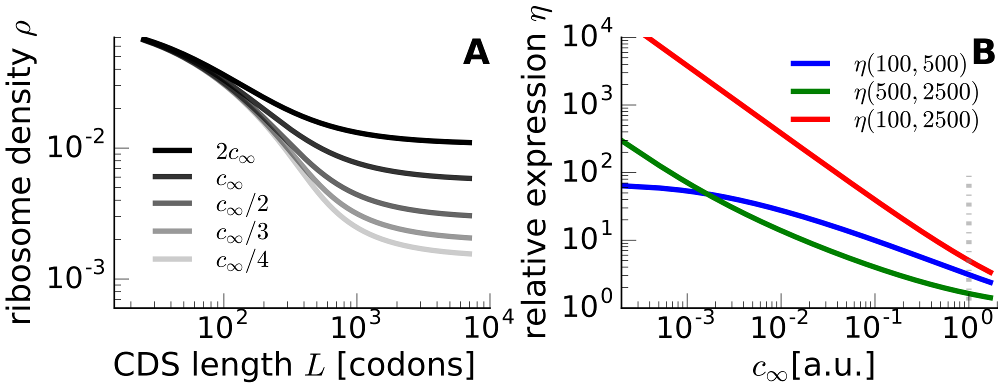

Figure S12. Equivalent to Fig.5, considering the diffusion of the two subunits as explained in Eq.(S15).

Supplementary Figure S13

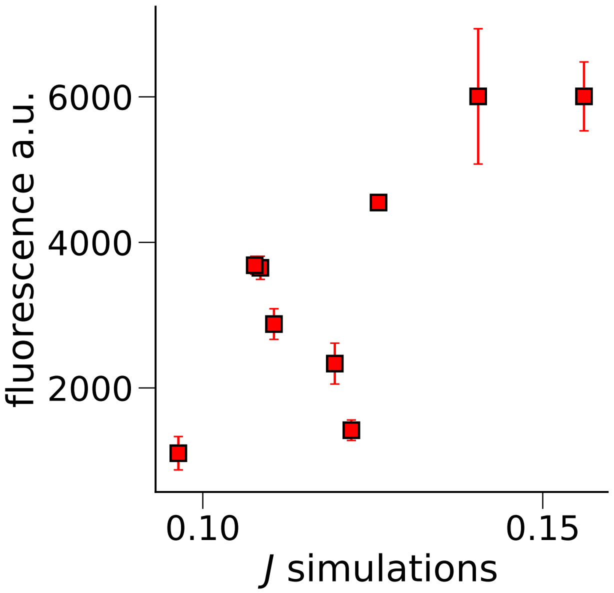

Figure S13. Comparison between the predicted protein production rates (J simulations, x-axis) and the experimental fluorescence of synonymous GFP sequences (y-axis) from Lanza *et al.*[15] (Pearson $r = 0.78$, p-value 0.014) with the parameters found for the Mackay dataset. Although we have used the model to extract the density-length relationship, Eq.(4) is also telling us how the initiation rate depends on the production rate J of its own transcript, which in this experiment changes since the codon usage of the synonymous GFP is varied. We obtain a good correlation between the outcome of the model and the observed experiment.
